# An anthracene based fluorescent probe for the selective and sensitive detection of Chromium (III) ions in an aqueous medium and its practical application

**DOI:** 10.3906/kim-2003-41

**Published:** 2020-08-18

**Authors:** Erman KARAKUŞ

**Affiliations:** 1 Organic Chemistry Laboratory, Chemistry Group, National Metrology Institute, (TÜBİTAK UME), Scientific and Technological Research Council of Turkey, Kocaeli Turkey

**Keywords:** Anthracene, thiophene, “turn-on” chemodosimeter, chromium (III) probe

## Abstract

An anthracene based fluorescent probe, integrated with thiophene moiety, exhibited selective and sensitive detection of chromium (III) ions over other metal ions. Its synthesis was achieved by simple mixing of two commercially available compounds, 2-aminoanthracene, and 2-thiophenecarboxaldehyde, in onestep without the needed complex purification process. The probe molecule ( **ANT-Th** ) offered exceptional features such as “turn-on” fluorescence response, low detection limit (0.4 μM), and fast response time (<1 min) via C=N bond hydrolysis. Also, a simple test paper system was developed for the rapid detection of chromium (III) ions with the naked eye.

## 1. Introduction

Among the heavy metal ions, chromium (III) ion has great impacts on the various systems such as chemical, biological and environmental systems. It is a crucial trace element in human nutrition to balance the “glucose tolerance factor”[1] and also acts as an essential role in the metabolism of proteins, nucleic acids, carbohydrates, and fats [2,3]. The National Research Council of United State has highly recommended taking 50–200 μg d^-1^ as an important amount of chromium (III) ion. Intake deficiency of chromium (III) ion can cause different types of diseases such as diabetes, cardiovascular disease, and impaired immune function [4,5]. However, the higher level of chromium (III) ion can lead to an abnormal effect on enzymatic activities and damage cellular structures [6,7]. Besides, chromium can also be revealed in the environment as a pollutant during some manufacturing processes such as tanning, steelworks, chromate, and chrome pigment production [8–10]. Therefore, sensitive, effective, and practical detection methods for chromium (III) ions are highly demanded.

Recently, fluorescence sensing methods have been very popular for the detection of trace metal analysis compared to the current traditional techniques such as atomic emission spectroscopy (AES), atomic absorption spectroscopy (AAS) and inductively coupled plasma mass spectroscopy (ICP-MS) [11–16]. Because they have manyadvantages such as operational simplicity, low-cost equipment, real-time detection, high sensitivity, and reproducibility. Some chromium (III) ion-selective chemical sensors have been developed by exploiting different types of fluorophore units including rhodamine [17–19], BODIPY [20,21], coumarin [22], anthracene [23,24]. However, some of these have still some drawbacks such as crosssensitivity toward other trivalent metal cations (especially Al^3+^, Fe^3+^) , slow response, low water solubility, and high detection limit. Therefore, it seems that there is a demand to develop new fluorescent sensors for chromium (III) ions which can overcome these problems.

Among the known fluorescent molecules, anthracene has attracted a great deal of our attention due to its unique properties such as its chemical stability, high quantum yield, simple structure, and easily chemical modification. Thus, anthracene and its derivatives have been used as molecular probes for the detection of pH, metal ions, and small organic molecules [25–32].

In this study, a facile one-step synthesis of a Schiff base probe ( **ANT-Th** ) is presented to detect chromiumions in aqueous media. This probe molecule offers distinct properties such as fast “turn-on” response, operability in the water medium, low detection limit, and the ability to differentiate chromium (III) from chromium (VI).

## 2. Experimental details

### 2.1. General methods

All reagents were purchased from the commercial suppliers (Sigma-Aldrich Chemie GmbH, Taufkirchen, Germany,and Merck KGaA, Darmstadt, Germany) and used without further any purification. 1 H NMR and 13 C NMR were measured on a VNMRJ 600 nuclear magnetic resonance spectrometer (Varian Inc., Palo Alto, CA, USA). Mass analyses were conducted with Thermo Q-Exactive Orbitrap device (Thermo Fisher Scientific Inc., Waltham, MA, USA). Fluorescence emission spectra were obtained using a Varian Cary Eclipse fluorescence spectrophotometer (Varian Inc.).

### 2.2. Preparation fluorescence emission measurement solutions

The stock solution of probe molecule **ANT-Th** (1 mM) was prepared in CH_3_CN and stock solutions of metal ion salts (20 mM) were prepared in triple distilled deionized water. During the measurements, the metal ion solution was added into the probe solution (2 mL) using a micropipette. For fluorescence measurements, samples were contained in 10.0 mm path length quartz cuvettes (2.0 mL volume). Upon excitation at 390 nm, the emission spectra were integrated over a range of 410–700 nm (both excitation and emission slit a width of 5 nm/5 nm). All measurements were conducted in triplicate at least.

### 2.3. Synthesis of ANT-Th

2-aminoanthracene (200.0 mg, 1.03 mmol) and 2-thiophenecarboxaldehyde (116.1 mg, 1.03 mmol) were mixed in 10 mL ethanol in the presence of catalytic amount (2–3 drops) acetic acid (AcOH). The solution mixture was refluxed for 6 h under the nitrogen atmosphere. The obtained solid was filtered and recrystallized in an EtOH-CH2 Cl2 mixture (3:1 v/v) to get the desired product **ANT-Th** as a dark green solid (67%). ^1^H-NMR (600 MHz, d-DMSO): δ ppm 9.00 (s, 1H, N=C-H), 8.55 (d, J = 12.6 Hz, 2H, Ar-H), 8.12 (d, J = 9 Hz, 1H,Ar-H), 8.06 (t, J = 8.4 Hz, 2H, Ar-H), 7.87 – 7.84 (m,2H, Ar-H), 7.74 – 7.73 (m, 1H, thiophene-H), 7.57 (d, J = 9 Hz, 1H, thiophene-H), 7.58 – 7.48 (m, 2H, Ar-H), 7.25 (t, J = 4.2 Hz, 1H, thiophene-H) . APT ^13^C-NMR (150 MHz, d-DMSO): δ ppm 157.0, 151.0, 145.7, 137.0, 134.9, 134.9, 134.5, 134.1, 133.1, 132.5, 131.5, 131.3, 130.9, 129.2, 129.1, 129.0, 128.6, 124.5, 121.4. HRMS: m/z: Calcd. for (C_19_H_13_NS) [M+H^+^]: 287.08470; found, 288.08493.

## 3. Results and discussion

**Scheme 1:**
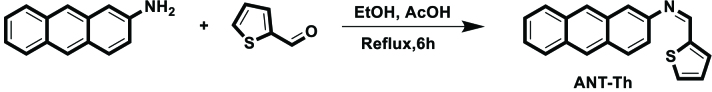
Synthesis pathway of ANT-Th.

The sensor molecule ANT-Th was obtained via facile one-step acid-catalyzed condensation reaction of readily available 2-aminoanthracene and 2-thiophenecarboxaldehyde without applying column chromatography for the purification process (Scheme 1). The chemical identity of ANT-Th was confirmed by nuclear magnetic resonance (NMR) spectroscopy and high-resolution mass spectroscopy (HRMS) techniques, as depicted in the Supporting Information (SI).

**Figure 1:**
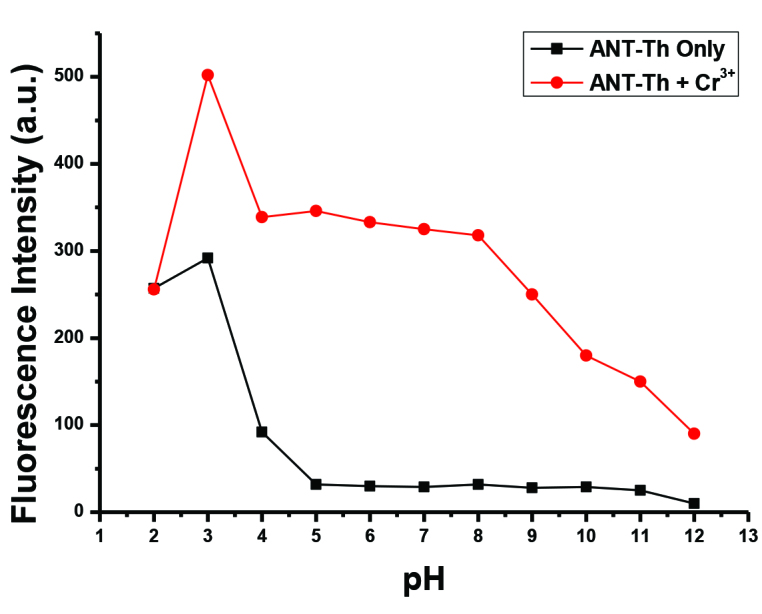
Effect of pH on the fluorescence intensity of **ANT-Th** (10 μM) in 6:4 CH_3_CN/HEPES in the absence (black line) and presence (red line) of Cr^3+^ (3.0 equiv.).

Firstly, it was investigated as the most efficient reaction medium for sensing events. Since **ANT-Th** structure hardly dissolves in completely aqueous media, the reasonable organic co-solvent is needed to increase the solubility, so the organic cosolvent should be miscible in water. Most proper solvent combinations were determined as EtOH-H_2_O and CH_3_CN-H_2_O mixtures yet sensing media was chosen as CH_3_CN:H_2_O due to the high fluorescence intensity changes. Then, all percent combinations (9:1, 8:2, 7:3, etc.) were tried and the best ratio was found as CH_3_CN:H_2_O (6:4 v/v) (Figure S1). However, it was realized that the pH of the sensing medium was effective for the detection of chromium (III) (Cr^3+^) ions. As can be seen in Figure 1, variance in the pH value led to obvious fluorescence intensity changes in the system. Thus, the pH of the sensing medium was adjusted to pH = 7.0 with HEPES buffer.

**Figure 2:**
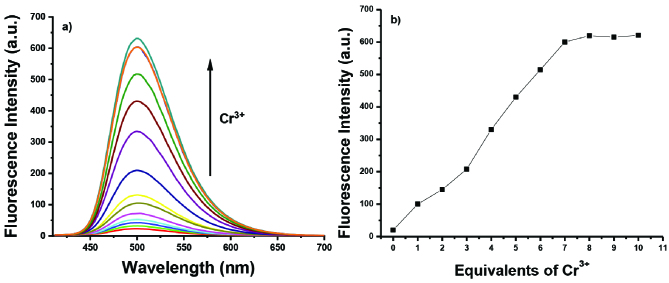
(a) Fluorescence titration spectra of ANT-Th (10 μM) in 6:4 CH_3_CN/HEPES at pH = 7.0 in the presence of Cr^3+^, (b) Fluorescence intensity changes depending on the number of equivalents of Cr^3+^ (mole equivalents = 0–10 equiv.).

As anticipated, the free **ANT-Th** was the fluorescence-off mode (ΦF = 0.01) due to the photoinduced electron transfer (PET) process in which the lone pair electrons on the nitrogen atom are transferred to the anthracene unit and as a result, its fluorescence ability diminishes. Upon the addition of the Cr^3+^ ions into the solution, a new strong emission peak appeared at 500 nm in a very short time (ΦF = 0.38). The emission intensity reached its maxima when 7 equiv. of Cr^3+^ ions were added, with a 30-fold enhancement (Figure 2). The detection limit (LOD) of the sensor molecule ( **ANT-Th** ) for Cr^3+^ ions was calculated as 0.4 μM (21 ppb) based on a signal-to-noise ratio (S/N = 3) (Figure S2). The spectroscopic response of **ANT-Th** was quite fast (<1 min), and complete saturation was observed after 5 min (Figure S3). Comparison of the ANTTh with some other Cr3+ sensitive fluorescent probes (Table S1) [22,24,33–42] clearly shows the superiority of this method. The LOD value of **ANT-Th** was lower than the other Schiff bases based fluorescent probe, as indicated in Table S1 [22,34,36,41,42]. Similarly, the response time of **ANT-Th** was quite rapid than other works [24,35,38–40,42]. Moreover, our probe can be the cheapest method as it involves a facile one-step reaction with commercially available chemicals without further complex purification process (Table S1).

**Figure 3:**
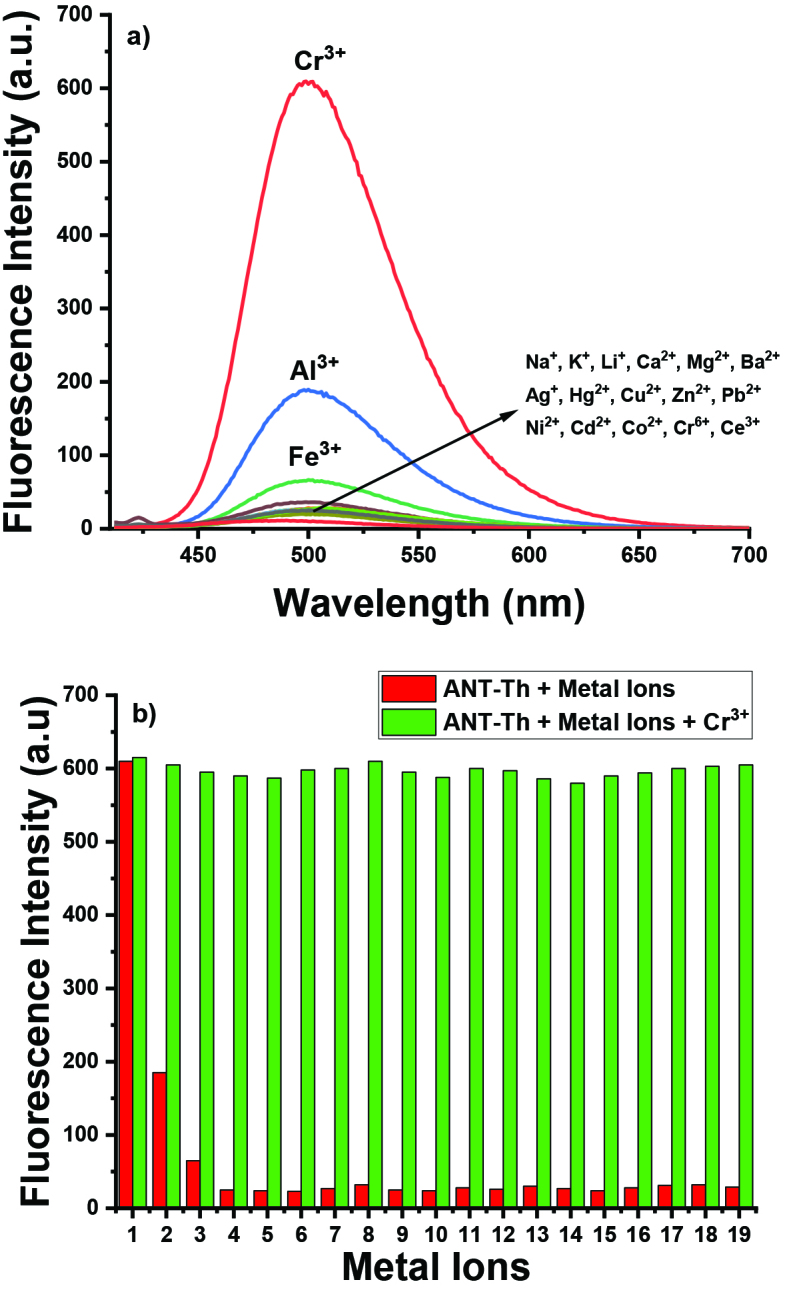
(a) Fluorescence intensities of ANT-Th (10 μM) in 6:4 CH_3_CN/HEPES at pH=7.0 in the presence of Cr3+ (5.0 equiv.) and other metal ions (25.0 equiv.), (b) Fluorescence intensities of ANT-Th (10 μM) in 6:4 CH_3_CN/HEPES at pH = 7.0 in the presence of Cr^3+^ (5.0 equiv.) and 25.0 equiv. other metal ions 1, Cr^3+^; 2, Al^3+^; 3, Fe^3+^; 4, Na^+^; 5, K^+^; 6, Li^+^; 7, Ca^2+^; 8, Mg^2+^; 9, Ba^2+^; 10, Ag^+^; 11, Hg^2+^; 12, Cu^2+^; 13, Zn^2+^; 14, Pb^2+^; 15, Ni^2+^; 16, Cd^2+^; 17, Co^2+^; 18, Ce^3+^; 19, Cr^6+^.

The selectivity study of ANT-Th was investigated by screening fluorescence intensity changes towards other metal ions including Na^+^, K^+^, Li^+^, Ca^2+^, Mg^2+^, Ba^2+^, Ag^+^, Hg^2+^, Zn^2+^, Pb^2+^, Ni^2+^, Cd^2+^, Co^2+^, Cr^6+^Fe^3+^, Al^3+^, and Ce^3+^. Fortunately, no significant alteration was observed in the presence of other metal ions except for Fe^3+^ and Al^3+^. These two metal species caused a similar fluorescence intensity change, but their intensities were relatively lower than that induced by Cr^3+^ even if their concentrations (250 μM) were much more than Cr^3+^ (50 μM) ions concentration (Figure 3a). It was further assessed the interference of other metal ions for the detection of Cr^3+^ by competitive experiments. The results showed that **ANT-Th** can properly detect Cr^3+^ ions in the mixture of other related metal ions (Figure 3b).

**Scheme 2:**
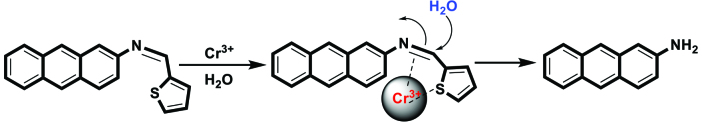
Proposed mechanism for the detection of Cr^3+^ ions.

To understand the sensing mechanism reversible or not, an excess amount of EDTA was added into the reaction medium containing **ANT-Th** probe and Cr^3+^ ions. Interestingly, the solution preserved its emission intensity, verifying that the sensing mechanism could be irreversible chemical reaction triggered by Cr^3+^ ions. The final product of the sensing event could be easily monitored by thin-layer chromatography (TLC). The appearance of the green emissive spot on the TLC plate with the same Rf value and the emission color of 2-aminoanthracene proved that the hydrolysis reaction of the **ANT-Th** probe occurred (Figure S4). With the help of HRMS analysis of the probe solution ( **ANT-Th** + Cr^3+^) revealed that a main molecular ion peak at m/z194.09 pointed out the exact molecular weight of the 2-aminoanthracene. As shown in Scheme 2, the sensing mechanism was proposed to proceed through the simultaneous coordination of Cr^3+^ ions with nitrogen atom on C=N moiety and S atom in the thiophene part. This event induced an attack of the water molecule to imine moiety leading the formation of hydrolysis product, 2-aminoanthracene (Scheme 2).

**Figure 4:**
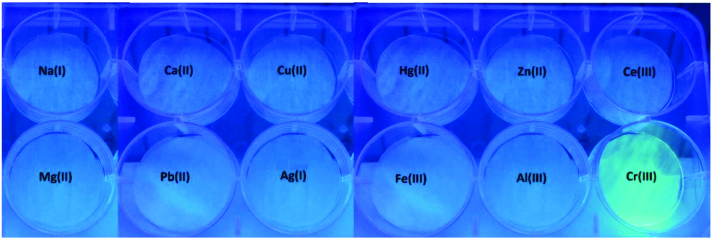
Detection of different metal ions (10μM) with ANT-Th (10μM) on circular test paper fluorescence images under UV light at 366 nm.

To investigate the practical application, **ANT-Th** (10 μM) in 6:4 CH_3_CN/HEPES solution at pH = 7.0 was added onto circular test papers and then they were dried in the air. Test papers did not show any emission under UV light at 366 nm without adding any metal species. As can be seen in Figure 4, only Cr^3+^ ions exhibited a “turn-on” response and gave brilliant green emission. Thus, it was concluded that these test papers can be used as a simple and fast tool for recognizing Cr^3+^ in a different area without using any sophisticated instrument.

## 4. Conclusion

In sum, it was exhibited the facile synthesis and spectroscopic behaviors of a “turn-on” type fluorescent probe (ANT-Th) for the detection of Cr^3+^ ions in an aqueous environment. The probe molecule demonstrated high selectivity with high fluorescence enhancement, low detection limit, and quick response time. Moreover, the monitoring of Cr^3+^ ions in solid-state with the naked eye as a practical application was successfully achieved.

Supplementary Materials
