# Supplementary material for: An anthracene based fluorescent probe for the selective and sensitive detection of Chromium (III) ions in an aqueous medium and its practical application
Source: Turk J Chem. 2020 Aug 18;44(4):941–9. doi: 10.3906/kim-2003-41 (PMC7751903; doi:10.3906/kim-2003-41)
Supplement: Supplementary file 1 — Supplementary Materials [file turkjchem-44-941-sup001.pdf]

Supporting Information

1. Determination of Quantum Yields

Fluorescence quantum yields of **ANT-Th** and **ANT-Th** + Cr^{3+} solutions were determined by using optically matching solutions of Rhodamine B (Φ_F=0.31 in water) as a standard. The quantum yield was calculated according to the equation;

$$\Phi_{F(X)} = \Phi_{F(S)} (A_S F_X / A_X F_S) (n_X/n_S)^2$$

Where Φ_F is the fluorescence quantum yield, A is the absorbance at the excitation wavelength, F is the area under the corrected emission curve, and n is the refractive index of the solvents used. Subscripts S and X refer to the standard and to the unknown, respectively.

2. Effect of Water Content

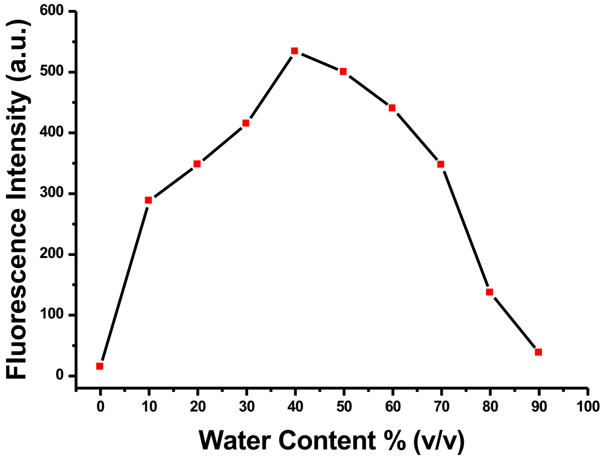

Figure S1 Effect of water content on the fluorescence intensity of **ANT-Th** (10 µM) in the presence of Cr^{3+} (60 µM) at pH= 7.0

3. Determination of Detection Limit of Cr^{3+}

The detection limit was calculated based on the fluorescence titration. To determine the S/N ratio, the emission intensity of **ANT-Th** (10 µM) without Cr^{3+} was measured by 10 times and

the standard deviation of blank measurements was determined. Under the present conditions, a good linear relationship between the fluorescence intensity and Cr^{3+} concentration could be obtained in the 0 – 10 µM. The detection limit is then calculated with the equation: detection limit = 3σbi/m, where σbi is the standard deviation of blank measurements; m is the slope between intensity versus sample concentration. The detection limit was measured to be 0.4 µM (21 ppb) at S/N = 3.

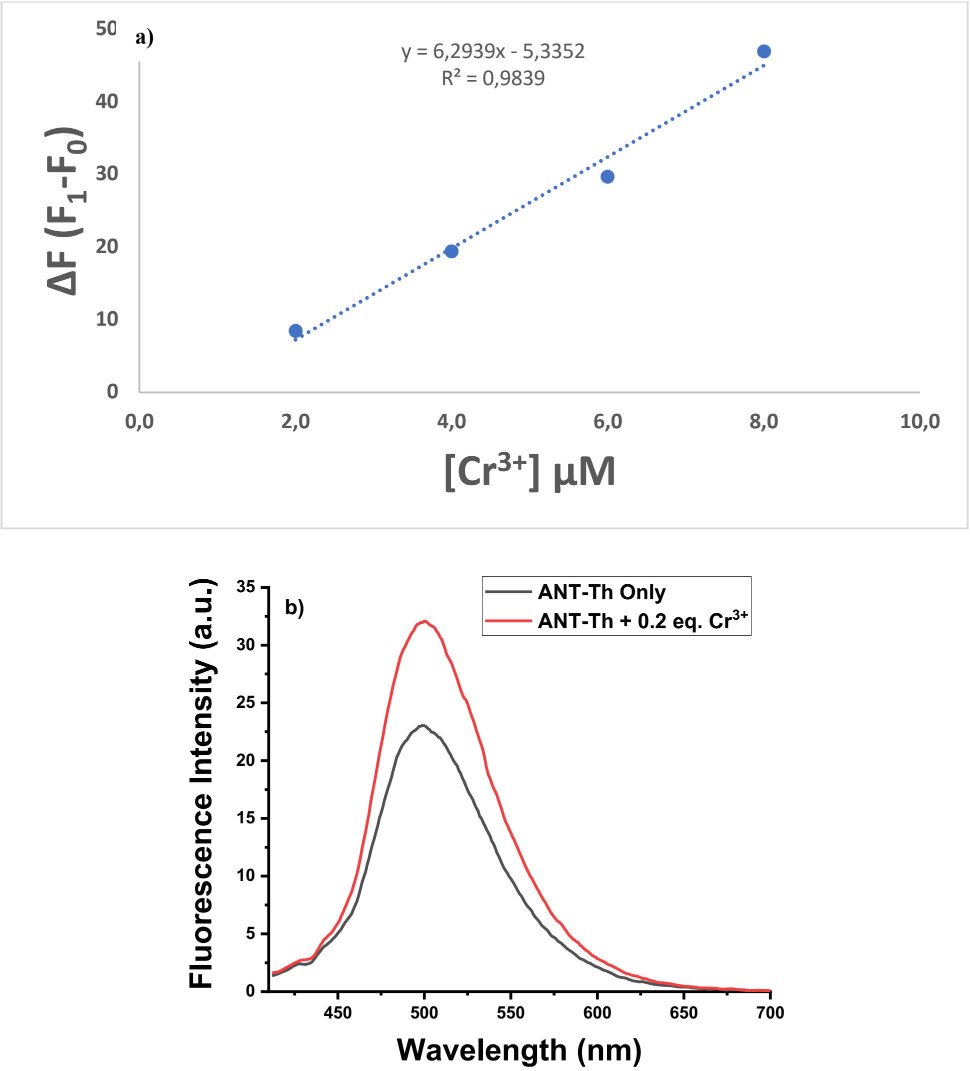

Figure S2 (a) Fluorescence changes of **ANT-Th** (10 µM) upon addition of Cr^{3+} (2.0 to 10.0 µM, 0.2 to 1.0 equiv.) (b) Fluorescence spectra of **ANT-Th** (10 µM) in the presence of Cr^{3+} (2.0 µM, 0.2 equiv.) in 6:4 CH_3CN/HEPES at pH=7.0

4. Time-dependent Fluorescence Change of ANT-Th

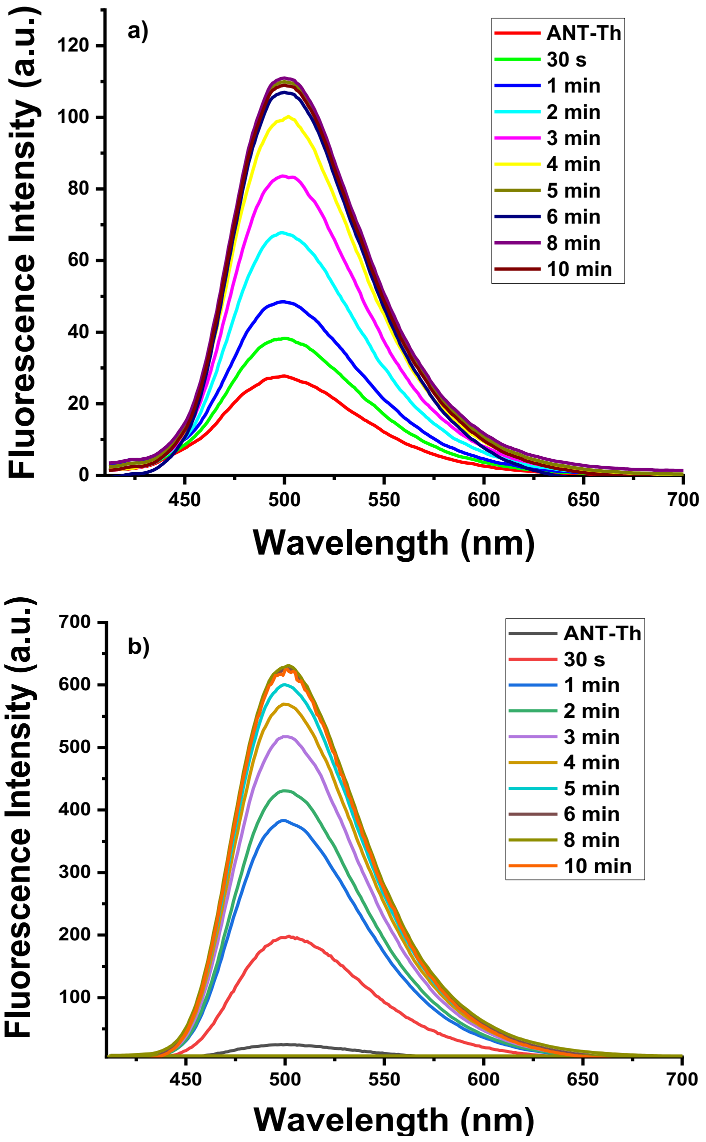

Figure S3 Time-dependent fluorescence change of **ANT-Th** (10 μM) in the presence of (a) 1.0 equivalent (b) 5.0 equivalent of Cr^{3+} measured in 6:4 CH_3CN/HEPES at pH=7.0

5. TLC image of the hydrolysis reaction of ANT-Th with Cr (III) ion

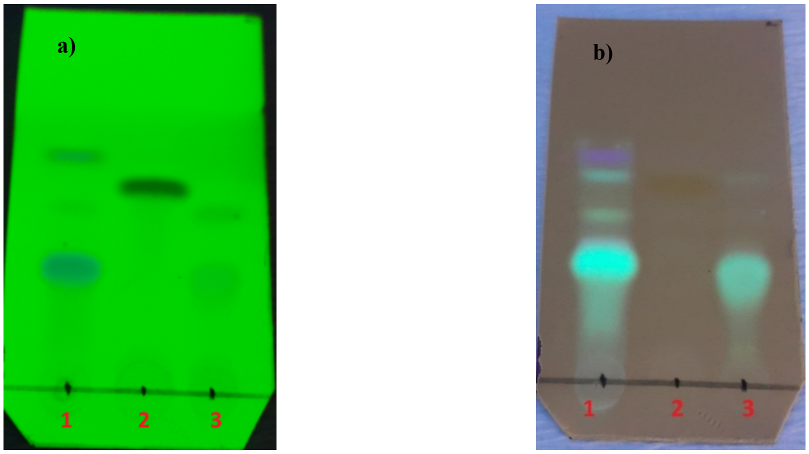

1. **2-Aminoanthracene Only** 2. **ANT-Th Only** 3. **ANT-Th + Cr^{3+}**

Figure S4. TLC image of the hydrolysis reaction of **ANT-Th** with Cr^{3+} ion under UV light **a)** 254 nm **b)** 366 nm

^{1}H-NMR and APT ^{13}C-NMR Spectra of ANT-Th

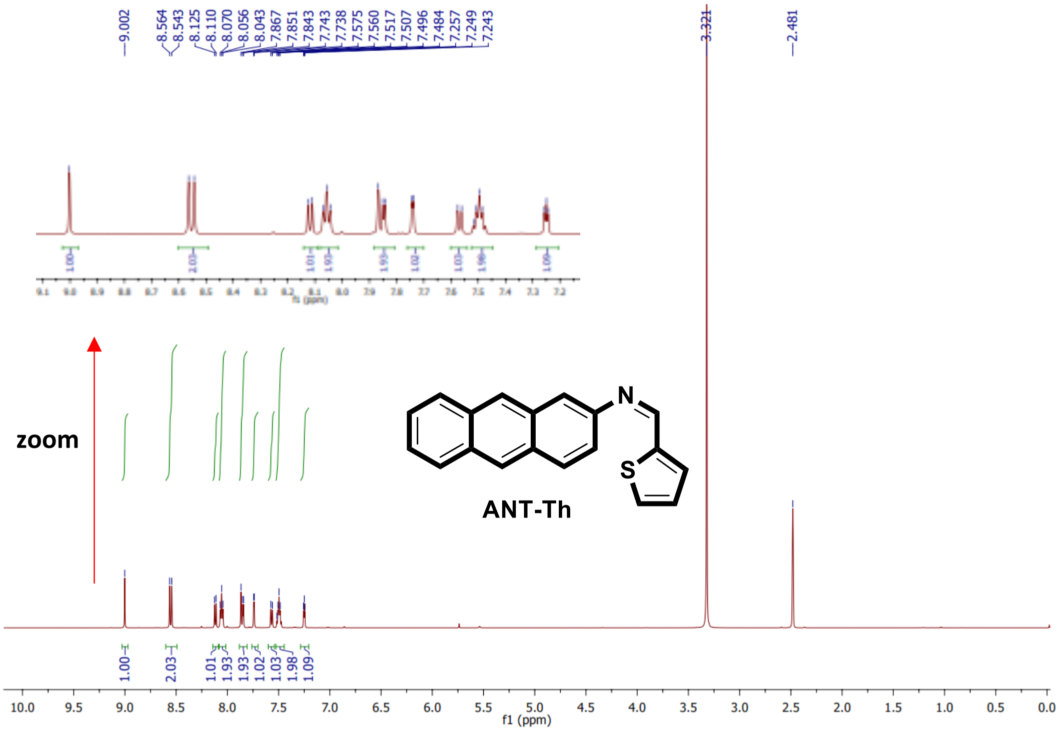

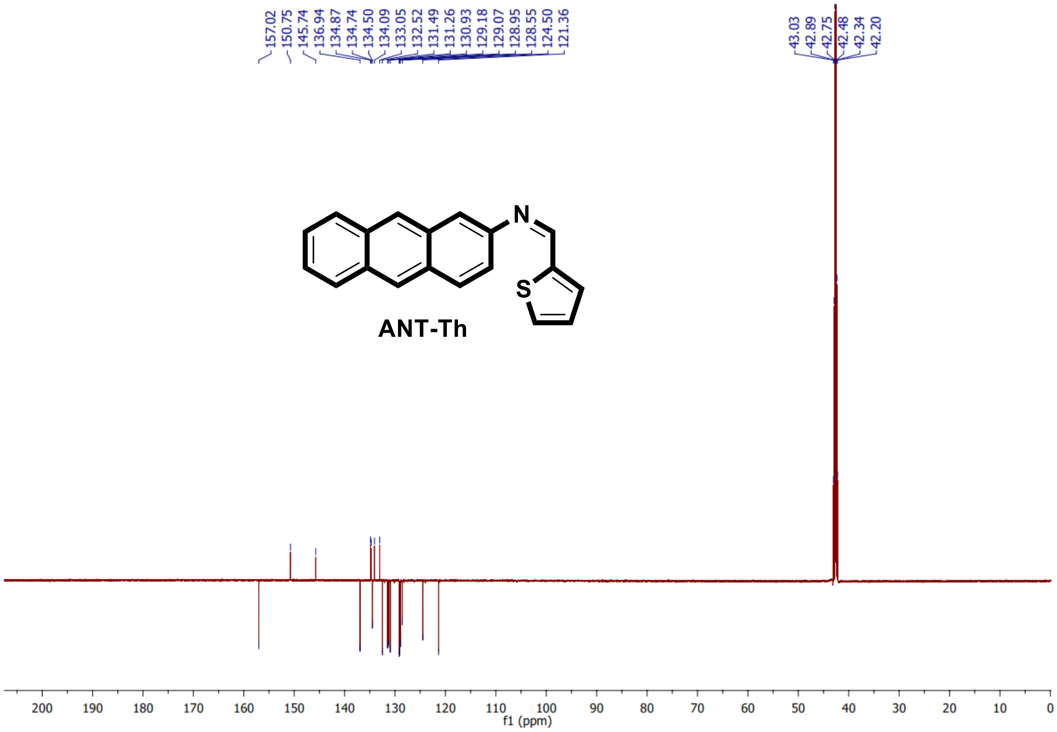

HRMS Spectrum of ANT-Th and Hydrolysis Product

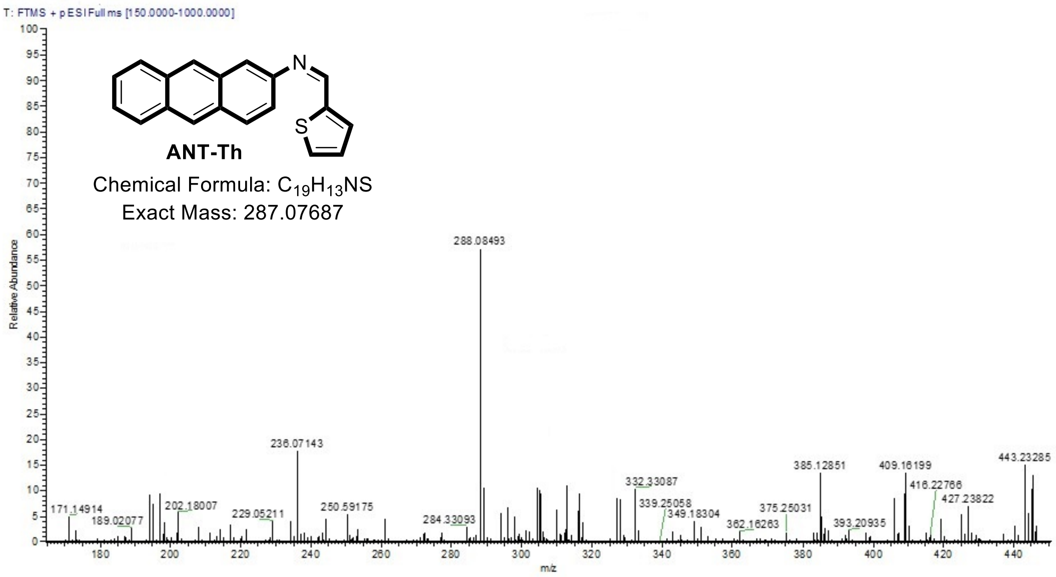

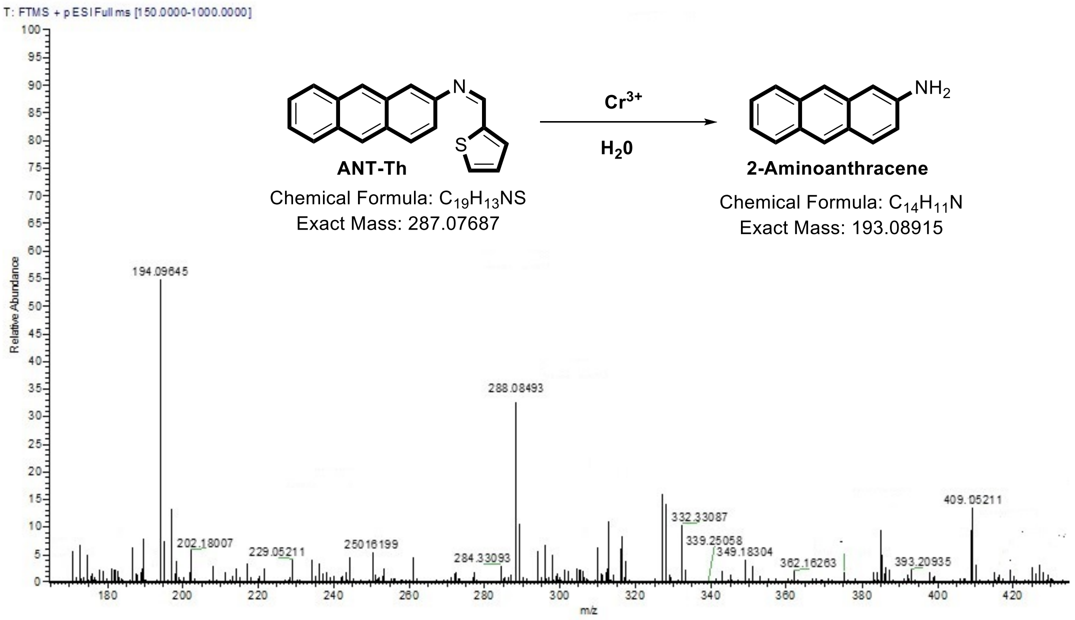
T: FTMS + p ESI Full ms [150.0000-1000.0000]
Cr3+
H20
ANT-Th
Chemical Formula: C19H13NS
Exact Mass: 287.07687
2-Aminoanthracene
Chemical Formula: C14H11N
Exact Mass: 193.08915
NH2
194.09645
288.08493
409.05211
332.33087
339.25058
349.18304
362.16263
393.20935
202.18007
229.05211
25016199
284.33093
Relative Abundance
m/z

Table S1. Comparison of the present method with other reported Cr^{3+} selective fluorescence probes

Compound	Synthesis Step	Mechanism	Solvent System	Response Time	LOD	Application	Ref. No
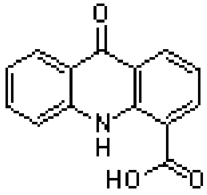	2 steps	Turn-off	9:1 (v/v) DMF: H_2O	NA	1 µM	NA	33
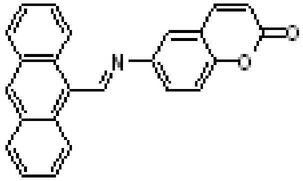	2 steps	Turn-on	9:1 (v/v) CH_3CN: H_2O	<2 min Saturation: 10 min	0.5 µM	YES	24
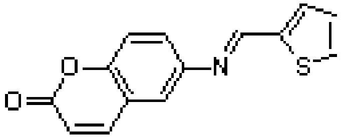	1 step	Turn-on	4:6 (v/v) CH_3CN : Buffer (HEPES) pH = 7.4	NA	1 µM	YES	22
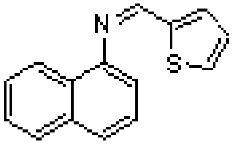	1 step	Turn-on	9:1 (v/v) MeOH: H_2O	NA	0.15 µM	YES	34
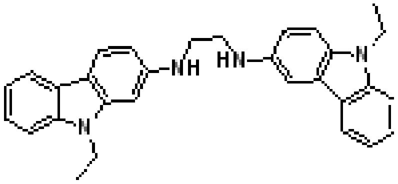	2 steps	Turn-off	9:1 (v/v) EtOH: Buffer (BR) pH= 7.4	2 min	0.1 µM	NA	35

Table S1. (Continued).

**Compound**	**Synthesis Step**	**Mechanism**	**Solvent System**	**Response Time**	**LOD**	**Application**	**Ref. No**
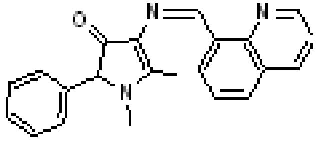	2 steps	Turn-on	4:1 (v/v) EtOH: H_2O	NA	1.5 µM	NA	36
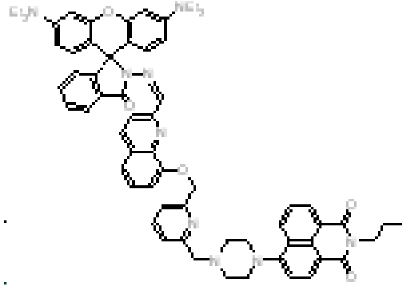	7 steps	Turn-on Ring-opening	1:1 (v/v) CH_3CN: Buffer (Tris-HCl) pH = 7.4	NA	0.2 µM	YES	37
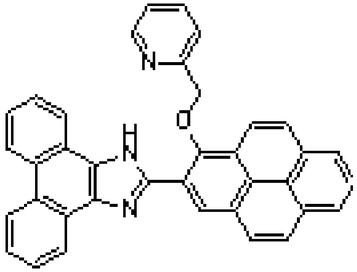	3 steps	Ratiometric	1:1 (v/v) THF: Buffer (HEPES) pH = 7.0	<10 min Saturation: 120 min	0.23 µM	NA	38

Table S1. (Continued).

Compound	Synthesis Step	Mechanism	Solvent System	Response Time	LOD	Application	Ref. No
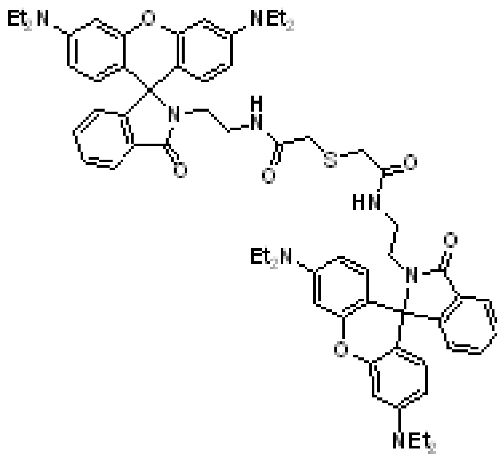 .	2 steps	Turn-on Ring-opening	3:1 (v/v) MeOH: Buffer (HEPES) pH = 7.2	<10 min Saturation: 60 min	0.38 µM	NA	39
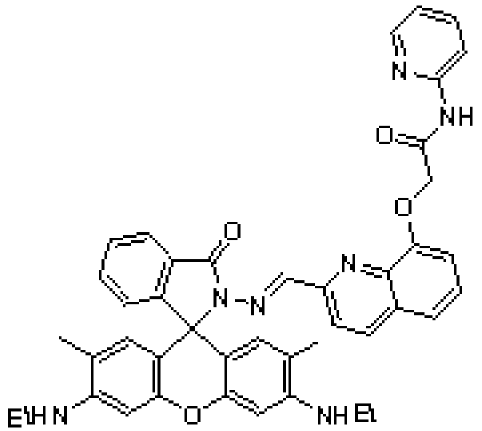 .	5 steps	Turn-on Ring-opening	9:1 (v/v) CH_3CN: Buffer (Tris-HCl) pH = 7.4	3 min	5.6 µM	YES	40

Table S1. (Continued).

Compound	Synthesis Step	Mechanism	Solvent System	Response Time	LOD	Application	Ref. No
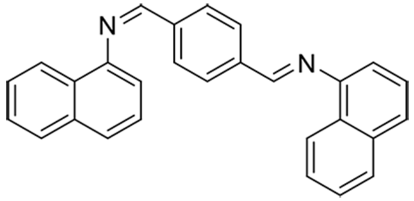	1 step	Turn-off	1:4 (v/v) CH_3CN: Buffer (HEPES) pH = 7.4	NA	3.92 µM	YES	41
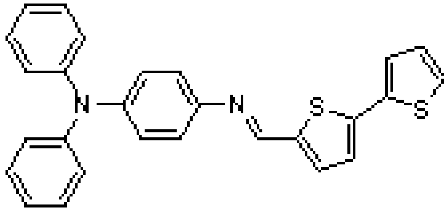	3 steps	Turn-on	1:1 (v/v) THF: H_2O	5 min	1.5 µM	NA	42
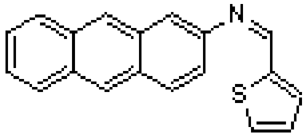	1 step	Turn-on Hydrolysis	6:4 (v/v) CH_3CN: Buffer (HEPES) pH = 7.0	<1 min Saturation: 5 min	0.4 µM	YES	This work
